# The effect of an occlusion-induced delay on braking behavior in critical situations: A driving simulator study

**DOI:** 10.1177/00187208221101301

**Published:** 2022-05-27

**Authors:** Joost C F de Winter, Mehdi Saffarian, John W Senders

**Affiliations:** 1 Cognitive Robotics, Delft University of Technology, Delft, Netherlands; 2 Department of Mechanical and Industrial Engineering, University of Toronto, Toronto, CA

**Keywords:** brake lights, brake control, occlusion, emergency braking

## Abstract

**Objective:**

To share results of an experiment that used visual occlusion for a new purpose: inducing a waiting time.

**Background:**

Senders was a leading figure in human factors. In his research on the visual demands of driving, he used occlusion techniques.

**Methods:**

In a simulator experiment, we examined how drivers brake for different levels of urgency and different visual conditions. In three blocks (1 = brake lights, 2 = no brake lights, 3 = occlusion), drivers followed a vehicle at 13.4 or 33.4 m distance. At certain moments, the lead vehicle decelerated moderately (1.7 m/s^2^) or strongly (6.5 m/s^2^). In the occlusion condition, the screens blanked for 0.4 s (if 6.5 m/s^2^) or 2.0 s (if 1.7 m/s^2^) when the lead vehicle started to decelerate. Participants were instructed to brake only after the occlusion ended.

**Results:**

The lack of brake lights caused a delayed response. In the occlusion condition, drivers adapted to the instructed late braking by braking harder. However, adaptation was not always possible: In the most urgent condition, most participants collided with the lead vehicle because the ego-vehicle’s deceleration limits were reached. In non-urgent conditions, some drivers braked unnecessarily hard. Furthermore, while waiting until the occlusion cleared, some drivers lightly touched the brake pedal.

**Conclusion:**

This experimental design demonstrates how drivers (sometimes fail to) adjust their braking behavior to the criticality of the situation.

**Application:**

The phenomena of biomechanical readiness and (inappropriate) dosing of the brake pedal may be relevant to safety, traffic flow, and ADAS design.

## Introduction

John Senders was a leading figure in Human Factors science. He is famous for his experiments on visual attention (Senders, 1964), which generated results that have stood the test of time (see Eisma et al., 2018 for a modern replication). In addition, Senders is known for his experiments on visual occlusion (Senders et al., 1967).

In his groundbreaking study, Senders (Senders et al., 1967) conducted experiments on a highway to examine what maximum speed drivers dare to drive when the occlusion time is systematically varied between 1 and 9 s while the viewing time remains fixed (between 0.25 and 1.0 s). Additionally, it was examined what voluntary occlusion time drivers adopt when systematically varying the vehicle’s speed between 22 and 60 mph, while again using a fixed viewing time. A second series of experiments were performed on a racetrack that included sharp curves and thus higher visual demands. The results of Senders’s experiments, combined with his theoretical modeling, elucidate how drivers respond to a lack of visual information and the minimum visual information required to drive.

The occlusion technique has now been applied in various driving-related tasks, including lane-keeping, cornering, braking, hazard anticipation, and secondary task performance (Akamatsu et al., 2013; Andersen et al., 1999; Borowsky et al., 2015; DeLucia & Tharanathan, 2009; Foley, 2008; Kiefer et al., 2006; Kujala et al., 2016; Saffarian et al., 2015; Van der Horst, 2004; Van Leeuwen et al., 2014). Here, we would like to share results of an experiment in which Senders was involved. Some preliminary results were published at the Driving Simulation Conference Europe (Saffarian, 2015).

The present study aimed to examine how drivers brake for different levels of urgency and different induced braking delays. How drivers react when confronted with a suddenly decelerating lead vehicle can be relevant for understanding the antecedents of accidents and traffic jams. A delay in braking may arise due to improper driving task prioritization, so that a two-stage response occurs, in which the driver may have a hunch that the situation is becoming critical but has not yet obtained the visual confirmation or the insight that braking is in fact necessary.

In the current study, occlusion was used not so much to withhold visual information, as in Senders’s landmark study, but rather to impose a waiting time. That is, participants were instructed to brake at any moment after the occlusion ended, which means that participants had less remaining time for braking in the occlusion condition compared to a control condition without occlusion. Additionally, in two of the three conditions, brake lights were removed to compromise the quality of visual information and to examine how participants brake if they have to rely on lead vehicle looming cues only.

## Methods

### Participants

12 participants (10 males, two females) with an Ontario class G driving license were recruited from the University of Toronto community. On average, the participants were 27.0 years old (*SD* = 6.8) and had obtained their first driving license 7.4 years ago (*SD* = 4.5). Four participants drove between 100 and 1,000 km/year, six participants drove between 1,000 and 10,000 km/year, and two participants drove between 10,000 and 100,000 km/year. Seven participants had previous experience driving a simulator. Participants were compensated with 30 Canadian Dollars. This research complied with the American Psychological Association Code of Ethics and was approved by the IRB at the University of Toronto. Informed consent was obtained from each participant.

### Driving simulator

The experiment was conducted using the NADS Minisim fixed-base driving simulator. The simulator presented the driving scene on three 42-inch plasma TVs, each with 1024 x 768 pixel resolution. An additional 19-inch screen acted as an instrument panel. The simulator recorded the data of the vehicle and control inputs at 60 Hz. Two speakers in the front provided stereo sound. Participants controlled the vehicle using the steering wheel, brake pedal, and gas pedal. Gear changing was automated.

### Experimental conditions and procedure

After arrival, participants read and signed an information/consent form. The form described the simulator controls and driving tasks. Next, participants completed a questionnaire that collected demographic and driving history data.

**Figure 1.**
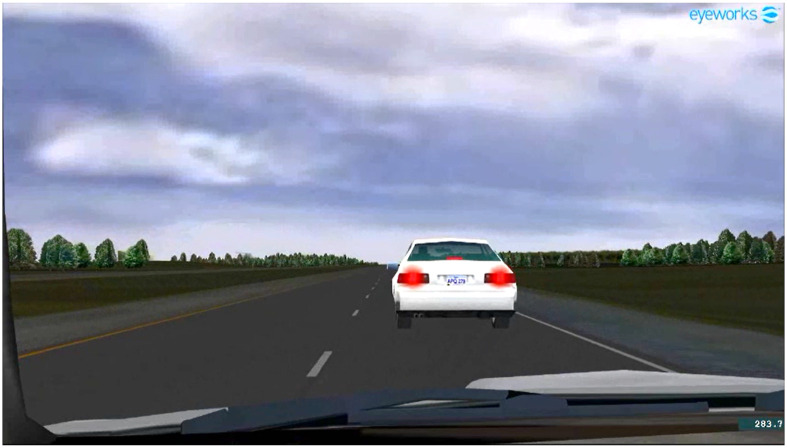
The participant’s view of the lead vehicle when the bumper-to-bumper distance was approximately 13.4 m, with brake lights on.

The experiment was of a 3 (visual condition) x 2 (following distance) x 2 (lead vehicle deceleration) within-subject design. The experiment consisted of three blocks in counterbalanced order. Each block lasted 22 min. In the *brake-lights* block, the lead vehicle’s brake lights were on when the lead vehicle braked (see Figure 1 for a screenshot). In the *no-brake-lights* block, the brake lights of the lead vehicle did not turn on. In the *occlusion* block, the simulator screens automatically blanked out for a short period as the lead vehicle started to brake, and the brake lights of the lead vehicle did not turn on.

The driving environment was a straight two-lane road with a lane width of 3.66 m. Participants first followed a lead vehicle at an instructed speed of 60 mph (96 km/h). The lead vehicle automatically maintained a set gap with respect to the participant’s vehicle. For half of the trials, the bumper-to-bumper distance was maintained at 13.4 m, and for the other half, it was maintained at 33.4 m. These following distances correspond to time headways (THWs) of 0.5 and 1.25 s, respectively. A THW of 0.5 s is regarded as an absolute limit adopted by a sizeable portion of drivers on highways, whereas a THW of 1.25 s is considered to be common (Fancher et al., 2001; Neubert et al., 1999; Taieb-Maimon & Shinar, 2001). In other words, the used headways, deceleration values, and occlusion durations (i.e., braking delays) correspond to normal versus critical conditions that may be encountered in real traffic.

After the car-following phase, the lead vehicle slowed down to 30 mph (48 km/h). In half of the trials, the deceleration involved hard braking at 6.5 m/s^2^ (2 s slowdown), and in the other half, it involved mild braking at 1.7 m/s^2^ (8 s slowdown). When the lead vehicle deceleration was large (6.5 m/s^2^), the occlusion lasted 0.4 s, whereas for the small deceleration (1.7 m/s^2^), the occlusion lasted 2.0 s. The simulator applied the deceleration of the lead vehicle with a 0.08 s delay to the event trigger. Research has shown that mean off-road glance durations range between 0.5 s (for glances at in-vehicle information systems such as the speedometer) and 1.5 s (for complex tasks, such as when reading street names or interacting with navigation devices; Birrell & Fowkes, 2014; Dingus et al., 1989).

**Figure 2.**
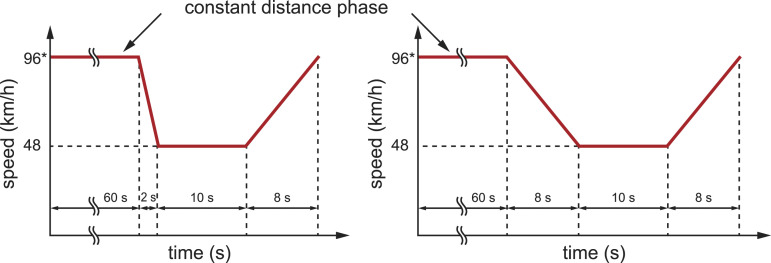
The lead vehicle speed scheme for large (left) and small (right) deceleration. *The 96 km/h speed during the 60 s constant speed phase varied between trials because the lead vehicle adapted its speed to the participants to achieve a constant headway. The speeds and times are approximate.

Each of the three blocks included four trials for each of the four braking conditions (1.7 m/s^2^ and 13.4 m, 1.7 m/s^2^ and 33.4 m, 6.5 m/s^2^ and 13.4 m, 6.5 m/s^2^ and 33.4 m). Hence, there were 16 braking trials within each block and 48 braking trials in total. The braking conditions within each block were presented in a fixed order (randomly generated before the experiment), which differed between the three driving blocks. Figure 2 shows the speed profiles of the lead vehicle for the two deceleration magnitudes.

A 6 min practice run preceded each driving block. During the practice run, participants gained experience with the braking tasks of the upcoming block. It consisted of one braking trial for each of the four braking conditions. The time between the braking trials was about 80 s.

### Instructions to participants

Participants were informed, in writing, that the goal of this study was to investigate how drivers use visual information to control their brakes. The instructions stated that the task was to drive at a speed of 60 mph while following the lead vehicle, that the lead vehicle maintained a set distance from the participant’s vehicle, and that the lead vehicle suddenly braked at certain moments. In addition, it informed the participants that when the lead vehicle braked, they had to slow down to avoid a collision. The form further stated that the participants should (1) try to control the brake force and avoid slamming the brake pedal, (2) drive swiftly but safely as in normal driving, (3) try to keep the vehicle centered in the right lane and not change lanes, (4) keep the right foot on the gas pedal before starting to use the brake. Participants were also informed that they would drive under three conditions in random order as follows:

(1) Brake lights: the brake lights of the lead vehicle are on; you can start braking at any time after the lead vehicle starts braking.
(2) No brake lights: the brake lights of the lead vehicle are off; you can start braking at any time after the lead vehicle starts braking, and
(3) Occlusion: when the lead vehicle starts to brake, the screen turns off for a short period; you should start braking at any time after the occlusion clears (i.e., when the screen turns back on); the brake lights of the lead vehicle are off.

The simulator recorded the brake pedal position using a potentiometer. A calibrated 100% pedal depression corresponded to pedal travel of 5 cm and a pedal force of about 150 *N*. The brake pedal force was approximately linear in the 0–100% depression range. Note that 100% was not the maximum physical depression that could be achieved; it was possible to press the brake pedal about 1 cm more deeply into the rubbers.

## Results

One of 576 trials was excluded because the participant was already braking when the lead vehicle started to decelerate. An additional 22 trials were excluded because the headway deviated more than 0.5 m from the target headway (13.4 or 33.4 m) due to the participant not speeding up enough.

**Figure 3.**
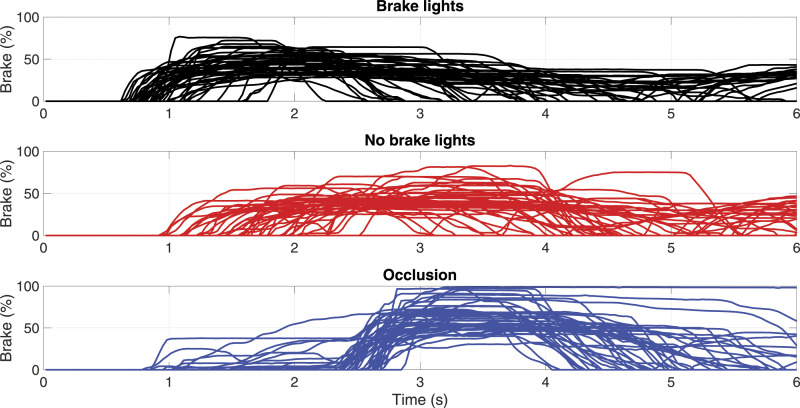
Brake pedal position in the 13.4 m, 1.7 m/s^2^ condition, for each of the recorded trials. In the occlusion condition, drivers were asked to brake after the 2 s-long occlusion had cleared.

Figure 3 shows the brake pedal position as a function of elapsed time for all trials of one of the four deceleration conditions (13.4 m, 1.7 m/s^2^). In the brake-lights condition (Figure 3, top), the brake response time was faster than in the no-brake-lights condition (Figure 3, middle). This finding can be explained by the fact that drivers have difficulty detecting the low deceleration of a lead vehicle based on looming cues only (Braunstein & Laughery, 1964; Park et al., 2001; Tharanathan & DeLucia, 2007).

**Figure 4.**
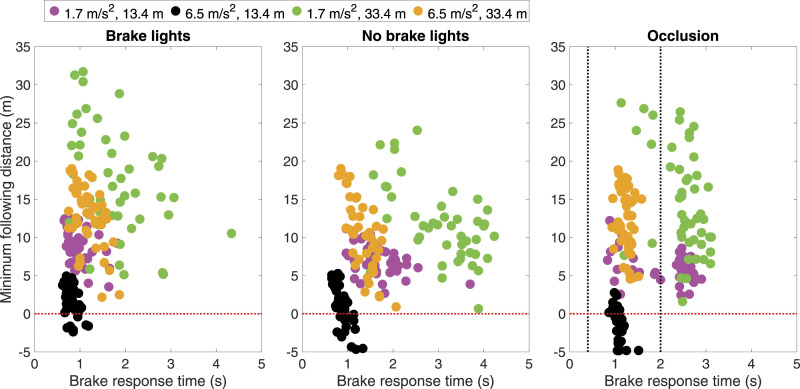
Scatter plot of brake response time and minimum following distance. Minimum following distances below the horizontal line were defined as collisions. Vertical dashed lines are drawn for the occlusion durations (0.4 s for 6.5 m/s^2^, 2.0 s for 1.7 m/s^2^).

A second finding is that drivers adapted their braking response. More specifically, in the 2-s occlusion condition (Figure 3, bottom), drivers braked late (in agreement with the task instructions) but compensated by braking harder. As a consequence of this adaptation, the minimum gap was hardly affected by the 2 s braking delay (as further illustrated by the green, magenta, and orange markers in Figure 4, showing a similar spread as the brake-lights condition). Some drivers pressed the brake up to 100%, which was an unnecessarily strong response for avoiding a collision. Presumably, these participants did not take the time to assess the deceleration of the lead vehicle (which was only moderate, at 1.7 m/s^2^) but slammed the brake, which caused a deceleration of 10 m/s^2^.

Of note, adaptation was possible only to a limited extent. In the worst-case scenario (13.4 m, 6.5 m/s^2^), a brief occlusion-induced braking delay (0.4 s, which is comparable to the duration of a glance at the speedometer, e.g., Dingus et al., 1989) substantially increased the number of collisions. More specifically, there were 67% collisions in the occlusion condition, compared to 16% in the condition with brake lights, and 30% in the condition without brake lights (see black markers lying below the horizontal line in Figure 4). The large number of collisions is explained by the fact that the pedal position was saturated at 100%, and therefore late braking is ‘lost time’ without the ability to compensate by decelerating more strongly.

A third phenomenon is that drivers showed preparatory behavior. In the occlusion condition, drivers were asked to withhold braking and keep their foot on the gas pedal. However, some participants braked or lightly touched the brake pedal before the occlusion had cleared (see Figure 3, bottom; and see the green and magenta markers lying left to the vertical line at 2 s in Figure 4, right), indicating biomechanical readiness. Additional figures depicting results for throttle position, brake position, vehicle deceleration, vehicle speed, and headway for the other urgency conditions can be found in the Supplementary Material.

## Discussion

Senders et al. (1967) was a pioneer in using occlusion to measure the attentional demands of driving. The present study used occlusion to induce a delay in the drivers’ braking response.

Several colleagues have told us that the present experimental design is unrealistic, as it does not resemble driver distraction on the road (a topic that Senders published about before; Senders, 2009). We obtained our results with alert drivers who could prepare for the upcoming threat, whereas drivers may not be alert in reality. On the other hand, it may be argued that if drivers engage in voluntary distraction, they exhibit some mental preparedness to brake (see Hanowski, 2011, who noted that certain secondary tasks prevent under-stimulation and could offer a protective effect against collisions).

Despite the questions that can be raised about realism, the present findings are illuminating in various ways. The experiment allowed for distinguishing between the effects of explicit cues (brake lights) and implicit cues (looming cues of vehicle motion), a topic that currently receives widespread attention in the context of external human-machine interfaces (eHMIs) for automated vehicles (Tabone et al., 2021). Moore et al. (2019) argued that eHMIs might not be needed as implicit cues usually provide sufficient information for road users in traffic. The present study, however, showed that explicit cues complement implicit cues, resulting in improved response times. The same principle seems to apply to eHMIs, including front brake lights or other displays mounted on the front or sides of the automated vehicle (e.g., Eisma et al., 2020; Monzel et al., 2021).

Furthermore, we showed that drivers adapt by braking harder if braking late. However, possibly because of a startle reaction, some drivers in the low-deceleration condition slammed the brake unnecessarily hard. This finding might be related to inappropriately hard braking behaviors in real traffic, after a visual or cognitive distraction. From traffic flow theory, it is known that small disturbances, even if not safety-critical, can propagate and cause disturbances in traffic flow, potentially causing a downstream traffic jam (e.g., Hoogendoorn & Bovy, 2001).

This study further showed that even a small braking delay of 0.4 s caused collisions in the most urgent condition, as adaptation was impossible due to saturated deceleration levels. This latter result could also be derived using classical mechanics^1^. Still, little literature exists that empirically demonstrates how drivers respond when being confronted with a highly critical situation, in which there is just enough time to avert a collision. In summary, the present study made clear that a collision results from the “impossibility to adapt”; that is, although more critical situations can be remedied by braking harder (i.e., adapting to the conditions at hand), there is a point when this is no longer possible as the vehicle’s deceleration limit is reached. Accordingly, the current results clarify why it is important to maintain sufficient following distance in traffic.

Finally, the present study illustrated the notion of biomechanical preparation, a topic that is of relevance to manual and automated driving. Weaver et al. (2021), for example, found that participants using a cooperative adaptive cruise control (CACC) system hovered their foot above the brake pedal in preparation for a merging event. In their study, participants especially did so in short-headway conditions, which is in line with our findings (see magenta markers in Figure 4, right). The topic of biomechanical readiness may also receive new interest in the area of graded warning systems (Bazilinskyy et al., 2018; Fagerlönn et al., 2012). For example, Zhang et al. (2019) presented a two-stage warning: a monitoring request, causing drivers to prepare to brake, followed by a take-over request. In a study examining drivers’ braking behavior in emergency situations, Prynne and Martin (1995) found that a high proportion of drivers applied two-stage braking “*with drivers rapidly depressing the brake pedal to the normal limit of depression (about a third of the full range available) and then depressing the pedal further to some lower position after they have thought about the situation.*” The occlusion of the current study also caused two-stage behavior in some drivers, with the drivers first braking lightly, followed by a step-like increase in brake pedal pressure. In our study, the duration of the first stage was controlled via visual occlusion.

In the works of Zhang et al. (2019) and Weaver et al. (2021), foot movement was annotated manually with the help of cameras (see also Wu et al., 2015), whereas, in our study, it was inferred from a light touch on the brake pedal. There may be potential to automatically measure foot movement (including hovering above the brake pedal) using image recognition software; such information could prove valuable input to driver assistance and driver monitoring systems.

It can be concluded that occlusion research can contribute useful demonstrations and possibly new insights, something which has been demonstrated by Senders before in his pioneering research (Senders et al., 1967). In his work, Senders always used small numbers of participants, but with many repetitions, an approach that is sensible for generating cumulative knowledge (Smith & Little, 2018). In the current study, a small number of participants (12) were exposed to as many as 48 braking trials, resulting in many collisions with the lead vehicle (while collisions are rare in reality). Occlusion in our study was not so much used to withhold visual information (because, after every occlusion, a braking action was necessary) but was a way to induce a delay in braking. Still, it can be expected that the occlusion created uncertainty amongst participants about how hard they had to brake.

Several limitations need to be considered. People underestimate the distance to a lead vehicle in the NADS Minisim (see Saffarian et al., 2015). Furthermore, drivers in a driving simulator usually brake harder and more abruptly than they do in on-road driving (Boer et al., 2000; De Groot et al., 2011; Shinar, 2017). Also, the driving scene was occluded completely. In actual driving, drivers may still extract valuable cues from the environment using peripheral vision (Lamble et al., 1999; Summala et al., 1998; Svärd et al., 2020; Terry et al., 2008), a topic studied by Senders himself in the context of distributed attention (Senders et al., 1955). Future research could include other combinations of independent variables, such as occlusion combined with brake lights, a wider range of occlusion durations, and different occlusion triggers (e.g., automatic triggering vs. triggered by the driver; Lansdown et al., 2004). Furthermore, future research could use occlusion not only when the lead vehicle brakes but also when the lead vehicle does not brake, to examine how drivers would respond in more surprising conditions.

## Supplemental Material

Supplemental Material - The effect of an occlusion-induced delay on braking behavior in critical situations: A driving simulator study Click here for additional data file. Supplemental Material for The effect of an occlusion-induced delay on braking behavior in critical situations: A driving simulator study by Joost C. F. de Winter, Mehdi Saffarian, John W. Senders in Human Factors: The Journal of the Human Factors and Ergonomics Society

## Note

1 If we extrapolate this and assume a non-adaptive driver who immediately presses the brake pedal to a constant position, the results can be calculated exactly. Suppose that two vehicles follow each other in the same lane, and assume that both vehicles are able to decelerate at a constant deceleration of 6.5 m/s^2^ (*a* = −6.5 m/s^2^). If further assuming that the brake response time of the following vehicle is 1 s (*t*_*b*_ = 1 s) and that the initial headway is 13.4 m, then the vehicles will collide 2.56 s (*t*_*c*_ = 2.56 s) after the lead vehicle starts braking, i.e., *t*_*c*_ = ((*a·t*_*b*_^*2*^)/2 – *D*_*0*_)/(*a·t*_*b*_).
