# Supplementary material for: The effect of an occlusion-induced delay on braking behavior in critical situations: A driving simulator study
Source: Hum Factors. 2022 May 27;65(7):1336–44. doi: 10.1177/00187208221101301 (PMC10845839; doi:10.1177/00187208221101301)
Supplement: Supplemental Material - The effect of an occlusion-induced delay on braking behavior in critical situations: A driving simulator study [file sj-pdf-1-hfs-10.1177_00187208221101301.pdf]

Supplementary Material

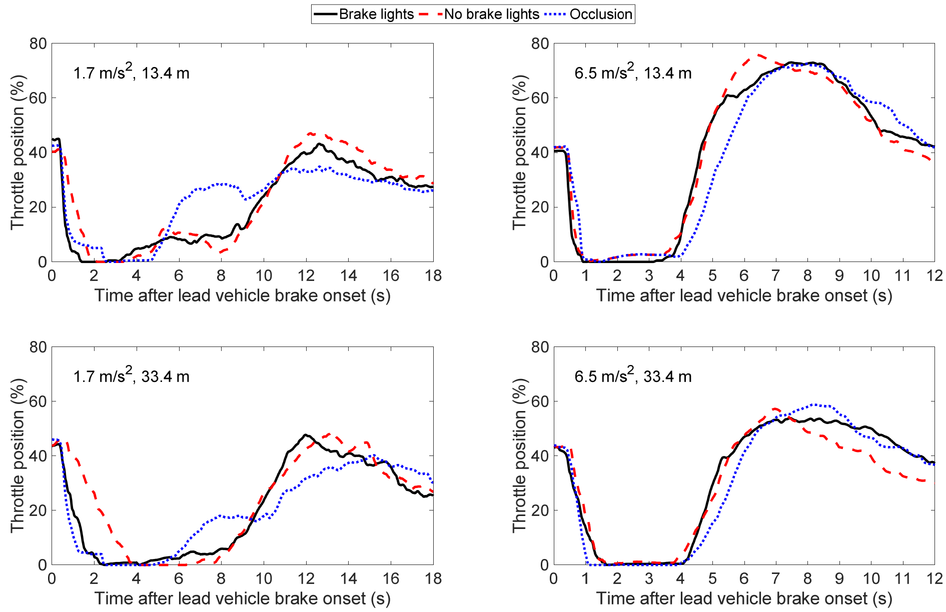

Figure S1. The mean throttle pedal position in the different braking conditions. Note that in the occlusion condition, the simulator screens blanked for 0.4 s (if 6.5 m/s^2) or 2.0 s (if 1.7 m/s^2).

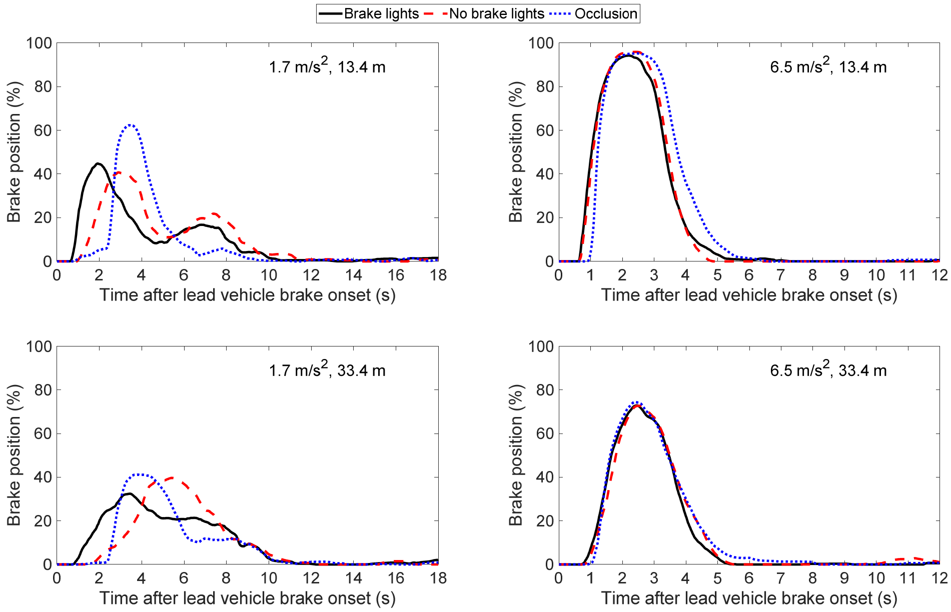

Figure S2. The mean brake pedal position in the different braking conditions. Note that in the occlusion condition, the simulator screens blanked for 0.4 s (if 6.5 m/s^2) or 2.0 s (if 1.7 m/s^2).

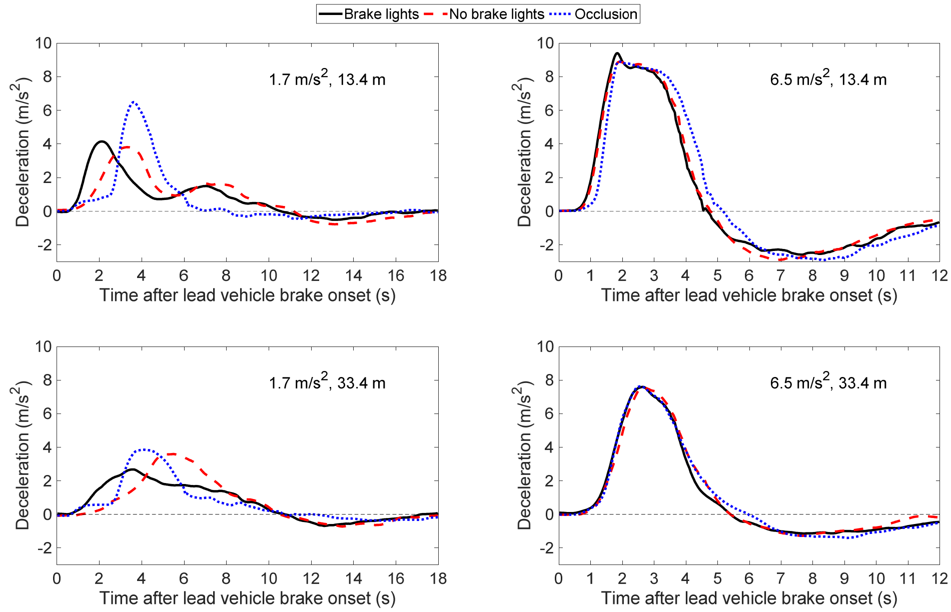

Figure S3. The vehicle deceleration in the different braking conditions. Note that in the occlusion condition, the simulator screens blanked for 0.4 s (if 6.5 m/s^2) or 2.0 s (if 1.7 m/s^2).

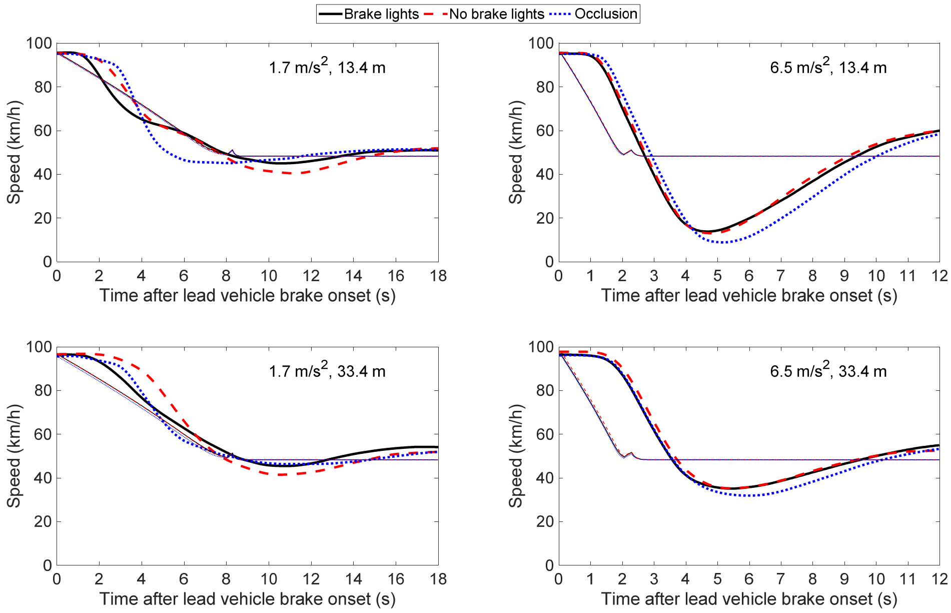

Figure S4. The mean participant's vehicle speed (thicker lines) and the mean lead vehicle speed (thinner lines) in the different braking conditions. Note that in the occlusion condition, the simulator screens blanked for 0.4 s (if 6.5 m/s^2) or 2.0 s (if 1.7 m/s^2).

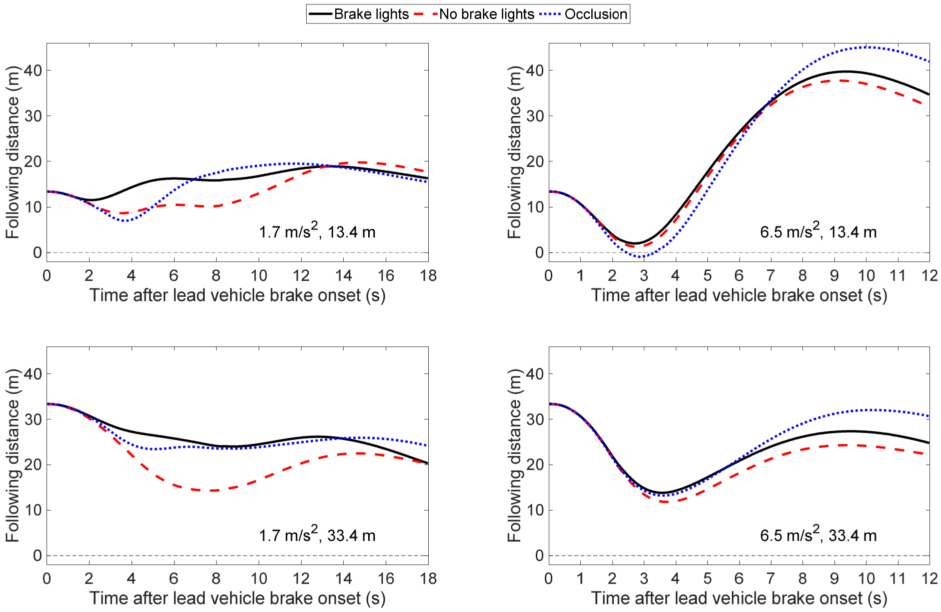

Figure S5. The mean bumper-to-bumper distance in the different braking conditions. Note that in the occlusion condition, the simulator screens blanked for 0.4 s (if 6.5 m/s^2) or 2.0 s (if 1.7 m/s^2).

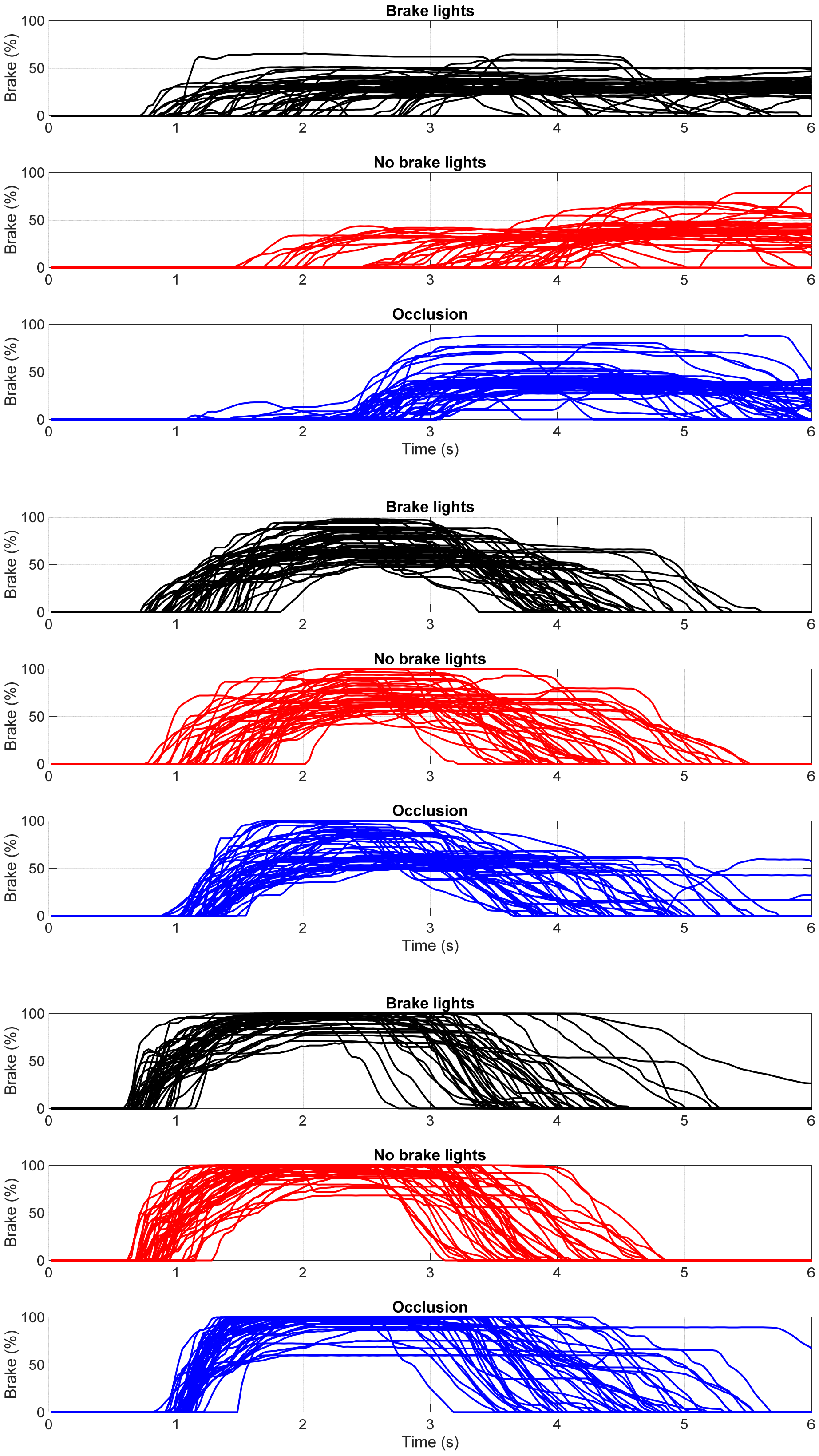

Figure S6. Brake pedal position in the 33.4 m, 1.7 m/s^2 condition (top), 33.4, 6.5 m/s^2 condition (middle), and 13.4 m, 6.5 m/s^2 condition (bottom), for each of the recorded trials.
